# Measuring anxiety disorder in bipolar disorder using EVestG: broad impact of medication groups

**DOI:** 10.3389/fneur.2023.1303287

**Published:** 2024-01-16

**Authors:** Brian J. Lithgow, Zahra Moussavi

**Affiliations:** ^1^Diagnostic and Neurosignal Processing Research Laboratory, Biomedical Engineering Program, University of Manitoba, Riverview Health Centre, Winnipeg, MB, Canada; ^2^Monash Alfred Psychiatry Research Centre, Prahran, VIC, Australia

**Keywords:** depression, electrovestibulography, biomarkers, anti-depressants, anti-psychotics, mood stabilizers

## Abstract

**Objectives:**

Anxiety disorder is present in approximately half of all bipolar disorder (BD) patients. There are neurologic bases for the comorbidity of balance (vestibular) disorders and anxiety. Our objective is to use electrovestibulography (EVestG), which is predominantly a measure of vestibular neural activity to not only quantitatively detect and measure comorbid anxiety disorder but also to quantitatively measure the impacts of anti-depressant, anti-psychotic, and mood stabilizer medication groups on anxiety measures in BD patients.

**Methods:**

In a population of 50 (24 with anxiety disorder) depressive phase BD patients, EVestG signals were measured. Participants were labeled depression-wise as anxious or non-anxious using standard questionnaires. Analyses were conducted on the whole dataset as well as on matched (age/gender/MADRS) and “modeled medication-free” subsets. Modulations of the low-frequency EVestG firing pattern data were measured.

**Findings:**

For BD, the main anxious minus non-anxious difference was the presence of an increase in spectral power proximal to 8–9 Hz, which was best attenuated by mood stabilizers.

**Novelty:**

This is the first study to use an oto-acoustic physiological measure to quantify anxiety disorder in BD wherein it appears to manifest as a peak proximal to 8–9 Hz which we hypothesize as likely linked to hippocampal theta.

## 1 Introduction

Studies suggest that anxiety disorder is present in approximately half of all bipolar disorder (BD) (1) patients. Thus, a clinically relevant quantitative and physiologically relevant anxiety test/measure beyond the standard questionnaire(s) would be of great interest. An improved understanding of the physiology behind differences between BD anxious and non-anxious states is also desired. A quantitative measurement of anxiety disorder may help in understanding its physiological bases, expediting targeted drug development. Most importantly, the main outcome could lead to a better identification and treatment of the symptomatology associated with comorbid anxiety disorder in patients.

There are neurologic bases for the comorbidity of balance (vestibular) disorders and anxiety (2). Electrovestibulography (EVestG) (3) is claimed to be a measure of vestibulo-acoustic activity (4) and has already been shown to quantitatively detect and monitor major depression as well as the depressive phase of bipolar disorder (5–7). EVestG detects spontaneous and driven mini field potentials predominantly from vestibulo-acoustic afferent activity and measures their change in the average field potential shape and the firing pattern of the detected field potentials as biomarkers useful in diagnosis, severity assessment, and treatment efficacy measurement. Previous studies have explored the measurement of depression in BD patients, with the final aim of distinguishing the depressive phase of BD from MDD (5–7). Those studies considered comorbid anxiety disorders cursively, concluding that some depression features might have been influenced by anxiety disorder (5–7). In the abovementioned BD study (7), in particular, the firing pattern feature changes were hypothesized to be affected by anxiety disorder.

The present study aims to examine the EVestG recordings of BD depressive phase patients, specifically focusing on the detected firing pattern changes observed with or without comorbid anxiety disorder, and model the groupwise effects of each of anti-depressant (AD), anti-psychotic (AP), and mood stabilizer (MS) medications on anxiety disorder.

We hypothesize those as follows: (1) EVestG firing pattern modulation features can robustly detect and measure comorbid anxiety disorder in a BD population and (2) the AD, AP, and MS medication groups have a significant effect on measurable anxiety and EVestG feature sensitivities.

The most important contribution and novelty of this study is on teasing out the effect of comorbid anxiety disorder in a BD depressive phase population using objective physiological measures (i.e., EVestG-derived features). This is important because anxiety disorder is often comorbid with neurological as well as mood (i.e., depression) disorders and can often be a hidden influencer of treatment efficacy. Therefore, teasing out the effect of comorbid anxiety disorder from depression and having a robust way to measure it might significantly improve our understanding of how specific symptom treatments can be applied more effectively. To the best of our knowledge, this is the first attempt to tease out comorbid anxiety disorder and the broad impacts of AP, AD, and MS medication groups on anxiety in a bipolar depressive phase population using an objective vestibulo-acoustic physiological measure.

## 2 Methods

### 2.1 Population data

The data for this study are collected from 50 individuals diagnosed by psychiatrists as BD (24 with comorbid anxiety disorder) (7). The presence of comorbid anxiety (or stress)-related disorders (ICD-10: F40, F41, F43) [in particular, generalized anxiety disorder (F41.1), social (F40.1) or specific phobias (F40.2), PTSD (F43.1), or panic disorders (F41.0) (8)] within this BD population were predominantly determined through the Mini-International Neuropsychiatric Interview assessment (9). The Montgomery–Asberg Depression Rating Scale (MADRS) (10) was applied as a measure of depression at the time of testing. Mania in BD was assessed using the Young Mania Rating Scale (YMRS) (11) and those with a score > 14 were excluded from this study. The Mini Mental State Exam (MMSE) (12) was applied to all participants as a measure of cognitive status. The EVestG data were recorded in the depressive/asymptomatic phase. Finally, two BD patients were not on AP, AD, or MS medication (Table 1 and Supplementary Table S1).

**Table 1:** BD participant summary demographics and assessments (μ ± SD).

**BD diagnosis**	**Age**	**Years since diagnosis**	**MMSE total**	**MADRS**	**YMRS**
**Asymptomatic or Mild [BD-R]**				
*n* = 32 (16 males), 13 with anxiety, MADRS ≤ 19	45.2 ± 13.8	14.8 ± 12.2	28.9 ± 1.4	7.4 ± 5.5	4.5 ± 4.3
**Mod-Severe [BD-S]**				
*n* = 18 (five males), 11 with anxiety, MADRS > 19	49.8 ± 12.4	17.4 ± 10.2	28.7 ± 1.4	28.7 ± 6.1	3.0 ± 4.1
**All [BD (BD-R & BD-S)]**				
*n* = 50 (21 males), 24 with anxiety	46.8 ± 13.7	15.7 ± 11.7	28.8 ± 1.4	14.8 ± 11.7	4.0 ± 4.3

The BD-R (reduced symptomatic) population has a MADRS score <19 and BD-S (symptomatic) MADRS score > 19 (see Supplementary Table S1 for detailed demographics).

### 2.2 EVestG recording and feature extraction

**Figure 1:**
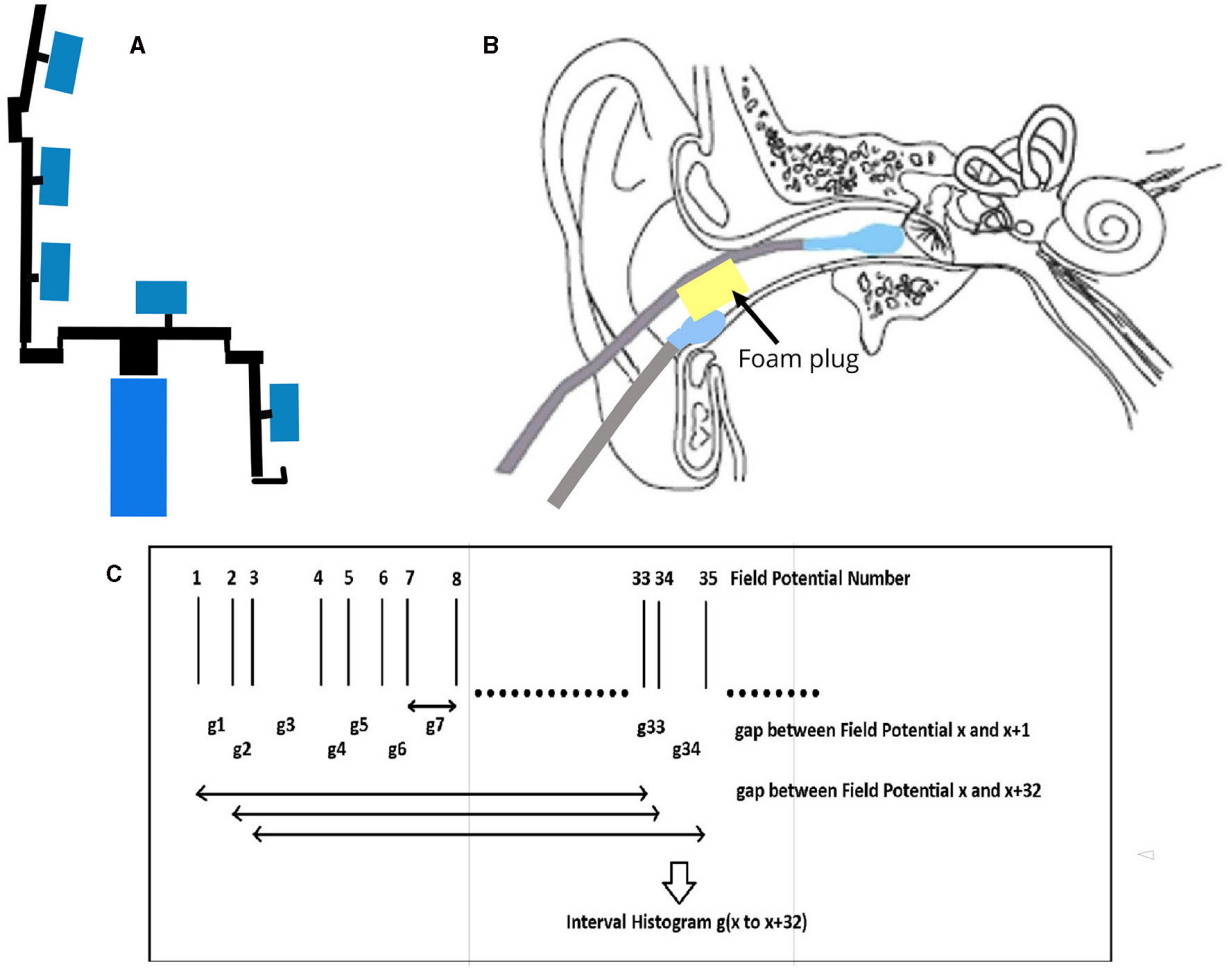
EVestG Recording. **(A)** Patient sits on chair in upright position. The response recorded with no motion and eyes closed. **(B)** Electrode connections. **(C)** IH33 timing information.

The EVestG recording methodology is detailed in (3, 7). In brief, the left- and right-side active recording electrodes are placed proximal to the ear drum (Figure 1B), reference electrodes are placed on the ear lobes or at the opening of the ear canals, and a common lead is placed on the forehead. With eyes closed, the subject responses were recorded in the stationary position (Figures 1A, B) while sitting upright. EVestG recordings were analyzed offline to detect the field potentials (FPs) using the NEER algorithm (3) that utilizes a complex Morlet wavelet analysis of phase to detect the FPs. The time intervals between any two detected FPs are calculated and presented as the firing pattern interval histogram, from which a histogram called IH_33_ (Figure 1C) is extracted. The IH_33_ histogram represents the interval histogram of every 33rd FP. As the experimentally determined gap between each FP is ~3.3 ms, by using each 33rd gap the focus is on the spectral content proximal to the hypothesized and potential links with the alpha band (8–13 Hz) and the lower end of vestibular efferent activity (5–7) looking for low-frequency modulations of the firing pattern proximal to 9 Hz (109 ms ~= 3.3 ms × 33). Importantly, hippocampal theta (4–12 Hz), which has been linked to anxiety circuits (13), also overlays in this frequency band.

We hypothesize that the stationary segment (no body motion also labeled as background) firing pattern interval histogram (IH_33_) will be impacted by anxiety disorder, i.e., without any physical tilting of the subject. Furthermore, these histograms have been previously postulated to, at least in part, be impacted by potential GABAergic changes purportedly present in anxiety disorder (5–7, 14, 15). Accordingly, the first step in using EVestG for detecting anxiety disorder will be to use only stationary (background) segments, which also have the advantage of minimal movement-related artifact corruption.

In summary, in this study, we will use the IH_33_ curve of the background (no motion) segments to detect the effect of comorbid anxiety disorder in BD depressive phase populations.

### 2.3 IH_33_ curve derivation

**Figure 2:**
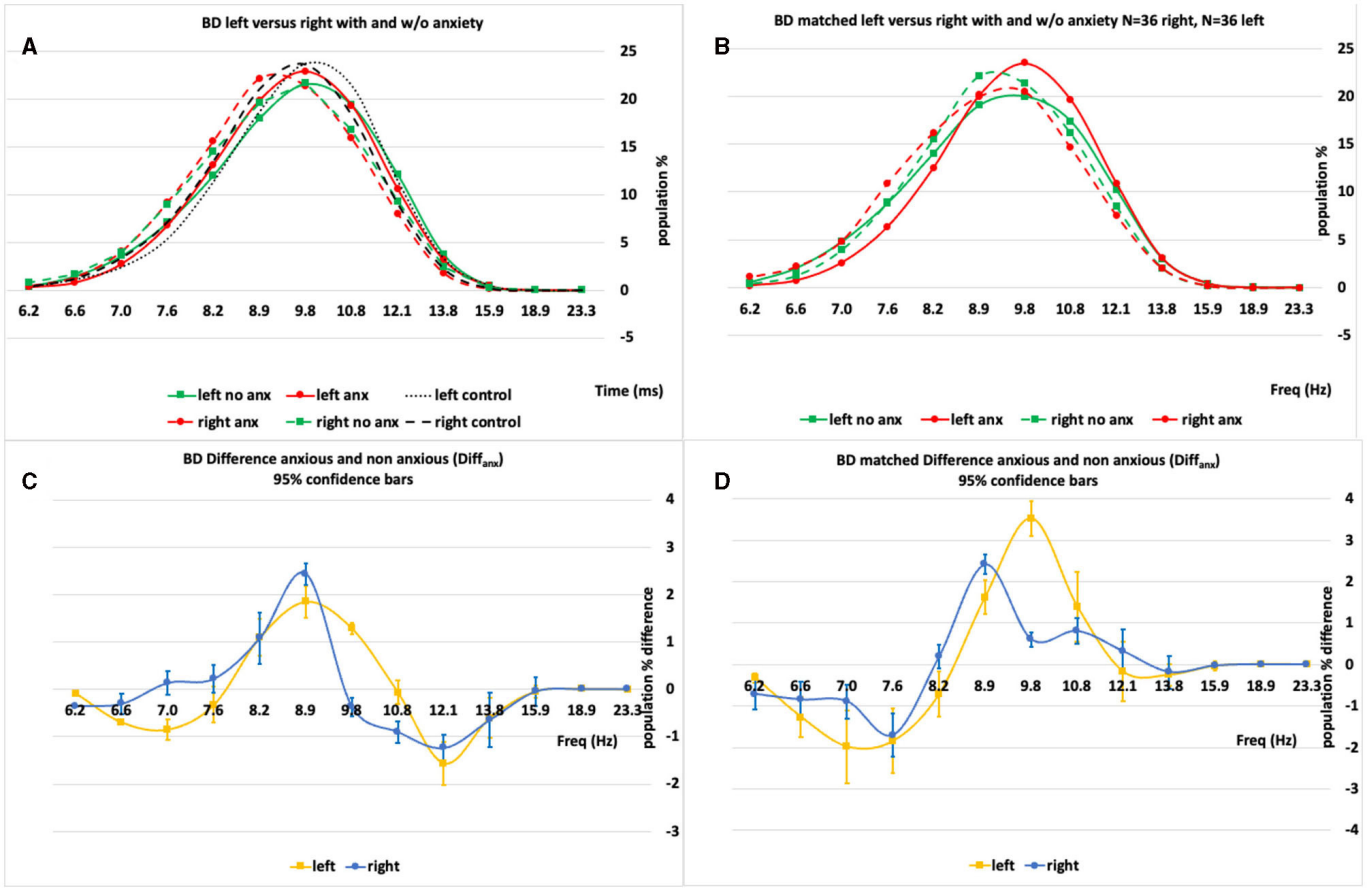
**(A, B)** An example set of IH33 plots for the BD “All” and BD “Matched” populations broken into their analysis subpopulations [anxious (anx.) and not anxious (no anx.)]. **(C, D)** The anxious minus non-anxious (Diffanx) IH33 plots highlighting the 9–10 Hz peak region as the largest Diffanx response. Left plots are “All” 50 BD patient right and left side data whilst right plots are “Matched” right and left side population responses after matching for age, gender and MADRS.

To derive the (e.g., Figures 2A, B) IH_33_ plots, the static recording segments analyzed herein were the average of five 1.5 s segments measured immediately before any applied whole-body tilt. These plots aimed to highlight potential low-frequency mediated modulations applied to vestibuloacoustic afferents.

**Figure 3:**
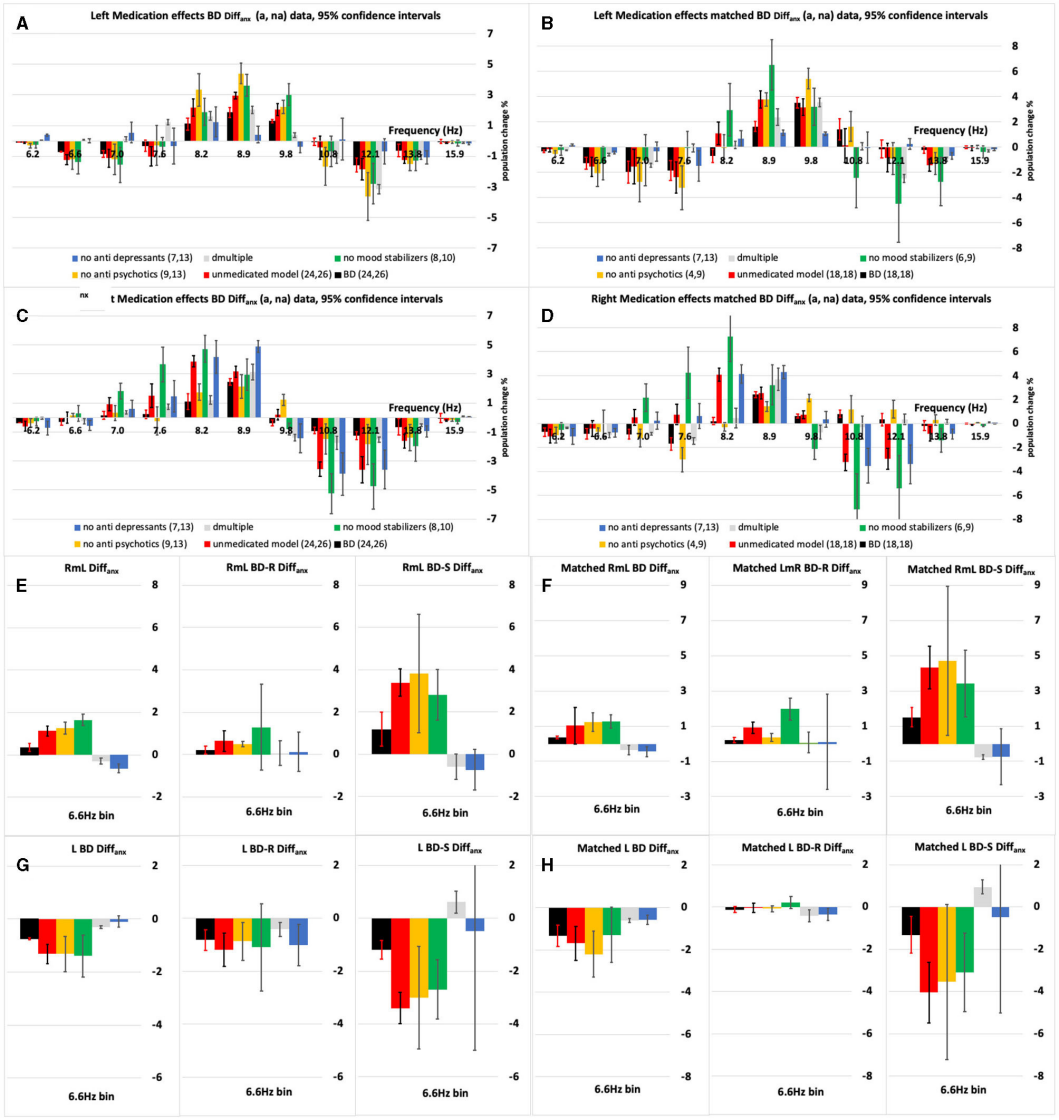
**(A–D)** The left and right-side anxious minus non-anxious (Diffanx) IH33 plots highlighting the effect of excluding one medication type at a time on the BD population Diffanx response. **(A, C)** Are “All” 50 BD patients and **(B, D)** are the “Matched” populations after matching for age, gender and MADRS. Medicated (black bars) and modelled Medication removed (red bars) responses show significant differences in the 8.2–9.8 Hz range. **(E, F)** Show there can be a significant right minus left (RmL) side Diffanx asymmetry in the 6.6 Hz bin. When the 6.6 Hz bin responses are broken into symptomatic (S–MADRS >19) and reduced symptomatic (R–MADRS <19) depressive severity subgroups it can be seen the S much more so than the R group is impacted by anxiety in the 6.6 Hz bin. **(G, H)** The left side **(L)** 6.6 Hz bin data is the dominant component in the right minus left **(E, F)** plots. For the modeled “medication removed” responses the *p*-values were adjusted. The leftmost pane in **(E–H)** is the R&S population, the middle panes are the R population and the rightmost the S populations.

The average IH_33_ anxious minus non-anxious (**Diff**_**anx**_) response curves before and after matching (age, gender, and MADRS) were compared for statistically significant differences with and “without” each medication group's (AD, AP, and MS) impact (Figure 3; see below and the Supplementary material for detailed medication compensation methodology).

### 2.4 Data analysis

The statistical tests were applied. In Figures 2C, D, 95% confidence interval significance is indicated on the Diff_anx_ plots by error bars. A selection of significantly different Diff_anx_ bins was used to derive classification features to discriminate anxiety disorder from non-anxiety disorder groups. Table 2 shows the definitions of these features based on the data from Figures 2, 3 (feature derivation is detailed below). Supplementary Table S2 shows the results of analyzing these features using analysis of covariance [(M)ANCOVA] or linear regression to determine each feature's robustness for identifying symptomatic (S), reduced symptomatic (R), and S &R populations of anxious from non-anxious patients. For the (M)ANCOVA, the fixed factors were class and gender; the covariates were age and MADRS scores, and the dependant variable(s) were the derived feature(s). For non-normal variables, linear regression and the Quade ANCOVA were applied with covariates/factors being derived feature(s), age, gender, and MADRS. The analysis was performed using SPSS V28 on the full dataset (“all”) and “matched” (age, gender, and MADRS) populations each with and without “medication effects removal” for the BD population. The matching subpopulations were selected by 1. first matching gender and then 2. best matching the MADRS and age combination while maintaining the largest possible population.

**Table 2:** Feature definitions and formation.

	**Definition (ROCmed/ROCnomed)**
F1_R_8.9 Hz	The right side 8.9 Hz bin (see Figures 2C, D). Right **Diff**_**anx**_ peak. **ROC** = 0.683/0.548
F2_L_6.6 Hz	The left side 6.6 Hz bin (see Figures 3A, B, E, F). S population focus. **ROC** = 0.543/0.705
F3_L_9.8 Hz	The left side 9.8 Hz bin (see Figures 2C, D). Left **Diff**_**anx**_ peak. **ROC** = 0.598/0.721
F4_RmL_6.6 Hz	The right minus left side asymmetry 6.6 Hz bin (see Figures 3E, F). S population focus. **ROC** = 0.599/0.955

ROC, receiver operating characteristic.

As all but two of the 50 BD patients were on medications, the entire population was grouped into those not on anti-depressants (notAD), those not on anti-psychotics (notAP), and those not on mood stabilizers (notMS) to determine the approximate averaged impact of each medication group. The not-on X responses were combined as per the methodology detailed in the Supplementary material, wherein a modeled “medication effect removed” Diff_anx_ response was estimated for each not-on-medication group and the combined not-on-medication group(s).

These R&S, R, and S left and right populations were all statistically analyzed as detailed above. For example, in the BD R&S left-side population, the effects of medication are shown in Figures 3A, B wherein each of the AP and MS but not AD medication groups decreased the Diff_anx_ 8.9 Hz region peak (see the Section 3 for detailed descriptions).

## 3 Results

### 3.1 Analysis of BD data IH_33_ patterns

Figures 2A, B show a set of left- and right-side IH_33_ plots for the BD (All, *N* = 50, 50 and Matched *N* = 36, 36) populations both with and without anxiety disorder. Figures 2C, D show that there are significant differences between those with and without anxiety disorder characterized predominantly by a peak in the anxious minus non-anxious (**Diff**_**anx**_) plot proximal to 8.9–9.8 Hz on both left and right sides. Although pattern-wise similar, the left- and right-side Diff_anx_ responses (Figures 2C, D) are significantly different.

Immediately apparent are the following: The BD-S&R Diff_anx_ response plots (Figure 3) show that, for all eight (left and right, all and matched, medicated, and “medication removed”) populations, at least, a small but significant (*p* < 0.05) spectral energy increase within the 8.2–9.8 Hz range when anxiety disorder is present. After considering consistency between “all” and “matched” populations, features F1 and F3 were generated from within these right and left significant regions (Table 2). There is also a smaller but significant decrease in Diff_anx_ spectral energy in the 6.6–7.0 Hz region, observed on the left but not the right side, for four (left, all and matched, medicated and “medication removed”) populations. After considering consistency between “all” and “matched” populations, features F2 and F4 were generated from these regions (Table 2).

These features of Table 2 were applied within a leave-one-out-cross-validated linear discriminant analysis (L1OCV), linear discriminant analysis (LDA), or naïve Bayes classifier (for non-normal variable distributions) in Table 3. After medication compensation, the feature F4 appears particularly suited to discriminate anxious from non-anxious groups. The classification analysis using “medication removed” features provides good classification (accuracy > 92% for S&R and R populations and >77% for S populations, Table 3). Classification without “medication removal” was only significant at 72–78% for the matched BD-R population. These results are now checked for statistical robustness.

**Table 3:** Classification accuracy results: Leave-One-Out-Cross-Validated Linear Discriminant Analysis (L1OCV LDA) for anxiety vs. non-anxiety.

**BD data**	**Best accuracy**		**Feature(s)**	**Best med. “removed” accuracy**		**Feature(s)**
S&R all	62%	[15, 11, 8, 16]	F3	92%	[23, 3, 1, 23]	F4
S&R m	72%	[12, 4, 5, 11]	F3	94%	[15, 1, 1, 15]	F4
R all	66%	[12, 7, 4, 9]	F1&F3	97%	[18, 1, 0, 13]	F3&F4
R m	78% (72%)	[7, 2, 2, 7] • [7, 2, 3, 6]	F1&F3 (F3)	100%	[9, 0, 0, 9]	F3&F4
S all^*^	78%, 44%	[5, 2, 2, 9] test	F1	100%, 66% (89%, 78%)	[7, 0, 0, 11] test • [6, 1, 1, 10] test	F3&F4 (F4)
S m^*^	79%, 47%	[7, 0, 3, 4] test	F1	93%, 66% (88%, 77%)	[7, 0, 1, 6] test • [7, 0, 2, 5] test	F3&F4 (F4)

^*^For non-normal distributions, the naïve Bayes classifier was applied [accuracy: training, test (50% test population, 10 folds)], m, matched; R, reduced symptomatic; S, symptomatic. [True +ve, False +ve, False –ve, True –ve]. All, unmatched entire population; Matched, age, gender, MADRS matching applied. F1–4 are classification features.

### 3.2 Statistical analysis

All the BD S&R Diff_anx_ responses without “medication removal” (Figure 3, black bars) show a small but a significant decrease within the 6.6 Hz range together with larger increases to at least one bin within the 8.2–9.8 Hz range. After “medication removal,” the “medication removed” populations (Figure 3, red bars) depict a significant Diff_anx_ spectral energy increase in at least one frequency bin within the 8.2–8.9Hz range.

A detailed statistical analysis based on Table 2 feature combinations is presented in Supplementary Table S2. In summary, from Supplementary Table S2 it can be presented as follows:

a. “**All**”. (Multi/uni)variate analyses showed for S&R (F3), R (F1 and F3), and S (F1 or F2) populations; these tested features were not able to provide significant anxious vs. non-anxious classification. Better matching of gender and age was required.
b. “**Matched**”. Only the reduced severity R population (F1 and F3) multivariate analysis produced a significant output: *[F1&F3; Multivariate: Wilkes-*λ = *0.517: F*_(2,11)_ = *5.133, sig* = *0.027*, η^2^ = *0.483, power* = *0.702, Univariate: F3, F*_(1,12)_ = *10.643, sig* = *0.007*, η^2^ = *0.470, power* = *0.849, F1, F*_(1,12)_ = *0.093, sig*=*0.765*, η^2^ = *0.008, power* = *0.059]*. Analysis showed that, without any medication compensation, feature F3 provides significant levels of anxious vs. non-anxious discrimination for the matched R population. Between-subject effects were not significant.
c. “**All** & **medication removed”**. S&R population: *[F4; Univariate: F*_(1,44)_ = *25.462, sig* = < *0.001*, η^2^ = *0.367, power* = *0.999]*. Analysis shows that the “medication removed” feature F4 provides a significant level of anxious vs. non-anxious discrimination. Between-subject effects for MADRS and F4 were just significant, p=0.05, indicating MADRS matching should be applied. R population: *[F3&F4; Multivariate: Wilkes-*λ*: F(2,25)* = *30.497, sig* = < *0.001*, η^2^ = *0.709, power* = *1.0, Univariate: F3, F*_(1,26)_ = *4.873, sig* = *0.036*, η^2^ = *0.158, power* = *0.566; F4, F*_(1,26)_ = *48.826, sig* = < *0.001*, η^2^ = *0.653, power* = *1.0.]*. Analysis shows that the “medication removed” features F3 and F4 provide a significant level of anxious vs. non-anxious discrimination (Supplementary Table S2b). S population*: [F3&F4; Linear Regression: F(5,12)*=*3.257, sig* = *0.044, Quade ANCOVA: F3, F*_(1,16)_ = *0.733, sig* = *0.405, t* = *0.856; F4, F*_(1,16)_ = *9.244, sig* = *0.008, t* = *3.040]*. Analysis shows the medication “removed” feature F4 provides a significant level of anxious vs. non-anxious discrimination (Supplementary Table S2c).
d. “**Matched & medication removed”**. S&R population. *[F4; Univariate: F*_(1,26)_ = *14.313, sig* = < *0.001*, η^2^ = *0.355, power* = *0.954.]* Analysis shows the “medication removed” feature F4 provides a significant level of anxious vs. non-anxious discrimination. R population: *[F3&F4; Multivariate: Wilkes-*λ*: F*_(2,11)_ = *17.168, sig* = < *0.001*, η^2^ = *0.757, power* = *0.997, Univariate: F3, F*_(1,12)_ = *18.788, sig* = < *0.001*, η^2^ = *0.610, power* = *0.987; F4, F*_(1,12)_ = *29.669, sig* = < *0.001*, η^2^ = *0.712, power* = *0.999.]* Analysis shows the “medication removed” features F3 and F4 provide a significant level of anxious vs. non-anxious discrimination (Supplementary Table S2b). S population: *[F3&F4; Linear Regression: F*_(5,8)_ = *1.758, sig* = *0.228, Quade ANCOVA: F3, F*_(1,12)_ = *0.626, sig* = *0.444, t* = *0.792; F4, F*_(1,12)_ = *8.904, sig* = *0.011, t* = *2.984.]*. Analysis shows that the “medication removed” feature F4 provides a significant level of anxious versus non-anxious discrimination (Supplementary Table S2c).

### 3.3 Medication effects

Only two of the BD population were not on anti-depressants (AD), anti-psychotics (AP), or mood stabilizers (MS), meaning a direct comparison of medicated and unmedicated was not possible. Figure 3 shows an analysis of the left and right “All” BD and “matched” BD Diff_anx_ responses after separately considering each of the not on AP, not on AD, and not on MS sub-populations. These sub-populations, for example, the not on MS (notMS), were made up of those on AP, AD, AD, and AP (AD^*^AP), and the not medicated (NM) groups (see Supplementary material for details of notAP and notAD definitions). The advantage of using the notXX groupings is that the sample size remains statistically meaningful. The Supplementary material also explains how the “medication removed” (red bar) responses are generated for use in Figure 3. The medicated responses (black bars in Figure 3) are made up of “All” of the (AD, AP, MS, MS^*^AP, MS^*^AD, AD^*^AP, AP^*^AD^*^MS, and NM) sub-population groupings. Except for Figures 2A, B, all figures were plotted with frequency (1/*t*) rather than the interval (*t*) for the horizontal axis.

#### 3.3.1 Combined S and R population

On the left side, the AP and MS medications reduce the 8.2–9.8 Hz Diff_anx_ peak, while the AD medications tended to act oppositely (Figures 3A, B). On the right side, the MS and/or AD medications reduce the 7.6–8.9 Hz Diff_anx_ peak (Figures 3C, D). MS consistently and significantly decreased this peak region on the left and right sides. The impacts of AD and AP medications were more asymmetric across left and right sides.

Figures 3E, F is the right minus left Diff_anx_ 6.6 Hz bin response and shows that depressive severity as well as left/right asymmetry can be a factor in the Diff_anx_ response. For the right minus, left Diff_anx_ response those classified as symptomatic (S–MADRS > 19) compared to reduced symptomatic (R–MADRS <19) presented not only with significantly larger 6.6 Hz Diff_anx_ bin values but larger medication effects. These effects support an AP and MS decrease and AD increase of this 6.6 Hz bin Diff_anx_ component and support the S population being more sensitive for this left-side dominated feature.

#### 3.3.2 Separate S and R populations

The R population response curves and most medication group responses are similar to those of the S&R population but generally larger in magnitude. This is because the S population responses can be broadly described as showing trends opposite in shape to the R curves more particularly on the right-hand side (Supplementary Figure S2). These broad-brush observations further support (as mentioned above for the left side 6.6 Hz bin) the Diff_anx_ (and medication group) responses also being a function of depressive severity and significantly so for the right-hand “All” and “Matched” and left-hand “Matched” side 8.2 and 12.1 Hz bins (Supplementary Figure S2).

For each of the classification features applied, using Figure 3 and Supplementary Figure S1, the impact of each medication group removal (orange, green, and blue bars) on the S&R, R, and S Diff_anx_ medicated response (Black bars) was evaluated (Table 4). Table 4 data show that, for feature F4 (right minus left Diff_anx_ 6.6 Hz bin), the impact of AP, AD, and MS medication groups is likely asymmetric, particularly for the S and perhaps S&R populations. Similarly, the impact of medication groups AD and AP on feature F3 (left Diff_anx_ 8.9 Hz bin) for the S&R population was significant.

**Table 4:** Significant (*p* < 0.05) impact of medications on the S&R, R, and S population Diff_anx_ responses.

	**All (S&R, R, S)**			**Matched (S&R, R, S)**		
	**notAD**	**notAP**	**notMS**	**notAD**	**notAP**	**notMS**
F1	Sig, X, X	X, Sig, X	X, X, X	Sig, Sig, X	Sig, X, Sig	X, X, Sig
F2	Sig, X, X	X, X, X	X, X, Sig	Sig, X, X	X, X, X	X, X, X
F3	Sig, X, X	Sig, X, Sig	Sig, X, X	Sig, X, X	Sig, X, Sig	X, X, X
F4	Sig, X, Sig	Sig, Sig, Sig	Sig, Sig, Sig	Sig, X, Sig	Sig, X, Sig	X, Sig, Sig

R, reduced symptomatic; S, symptomatic; Sig., significant; X, not significant.

### 3.4 Depressive severity

We now briefly further consider whether there is a different BD-R vs. BD-S response to anxiety disorder. From Figures 3E, F and from feature correlations with MADRS, it can be seen that features F2 and F4 (Pearson, F4/F4MR, 0.081/-0.418^**^, F2/F2MR, 0.228/0.265; Non-parametric Spearman-rho, F4/F4MR, 0.129/-0.476^**^, F2/F2MR, 0.282^*^/0.256, MR = *medication “removed”)* are correlated with MADRS and significantly larger for the symptomatic-S rather than reduced symptomatic-R population (^**^significant at *p* < 0.01, ^*^significant at *p* < 0.05, MC = “medication removed”).

## 4 Discussion

In BD, it takes only one manic symptom during depression to spark anxiety (16). There are significant and measurable differences between anxiety disorder and non-anxiety disorder BD patients (Figure 2). For the BD and BD-R anxious populations, the most obvious difference was an increase in spectral power (Figures 2, 3; Supplementary Figure S1) within the 8.2–10.8 Hz range. This spectral power was increased (in the 8.2–8.9 Hz range) after “medication removal” (black c.f., red bars in Figure 3). These data support, though not significantly at all frequencies in the above range, MS and AP or AD medications, overall, suppressing these Diff_anx_ frequency components (Figure 3). Interestingly, for the BD-S population, after “medication removal,” there was a significant (though not at all frequencies) decrease in spectra energy proximal to 8.2–8.9 Hz, i.e., opposite to the decrease seen for the BD-R population (Supplementary Figure S1).

It is known that MS's such as S-valproate can improve anxiety symptoms in BD perhaps because it acts as a potential inhibitor of GABA metabolism and enhancer of its production (17). Moreover, APs such as quetiapine have been shown to reduce anxiety (18). Finally, when treating mixed states BD, it is common to consider reducing ADs (19). These three medication group findings generally support the left-side S&R medication effects presented in Figures 3A, B in the 8.2–8.9 Hz range. The right-side plots (Figures 3C, D) only support the MS group findings.

There is frontal asymmetry in the theta band in BD depression (20). There is also evidence of asymmetry in the caloric vestibular response in depression (21, 22). Furthermore, there are neurologic bases for the comorbidity of balance (vestibular) disorders and anxiety (23). Supporting these three observations, it was noted in the statistical analysis that the asymmetry measure that the right minus left feature F4 was particularly sensitive to the Diff_anx_ “medication removed” response. A future study might explore asymmetry being a marker for anxiety disorder in newly diagnosed depressives.

There is evidence of EEG alpha band activity being successfully applied to predict depressive severity (24). It is also noted that the medicated vs. non-medicated response differences are mostly significant in this frequency range. These findings support depressive severity acting as potentially confounding influences on the effects medication groups may have on the Diff_anx_ plots of Figures 2, 3 and Supplementary Figure S1. Additionally, low vs. high theta current density in the frontal cortex and rostral anterior cingulate has been associated with response and non-response, respectively, to ADs (25). Furthermore, anxiety has been shown to be correlated with both alpha and theta bands (26). These findings also support both anxiety disorder and depressive severity acting as potentially confounding influences on the effects medication groups may have on the Diff_anx_ plots of Figures 2, 3 and Supplementary Figure S1.

The analysis of both the BD-R ‘Matched” and BD-R “All” medication compensation was deemed particularly important given that the presence of anxiety in women is approximately twice that observed in men (27), and this was reflected in our overall study numbers.

### 4.1 GABA in BD

There appears to be a strong association between BD and polymorphisms at the level of GABAA receptor subunit genes (28). The strongest evidence that GABAergic deficits may contribute to BD depressive disorders is the observed reductions in GABA levels in plasma and cerebrospinal fluid or the resected cortical tissue (28). In BD, GABA is argued to be more reduced (28), potentially reducing the spontaneous discharge of the vestibular afferents. Disruptions of inhibition associated with GABAergic activity might also have a vascular basis (29). Additionally, activation of GABA_B_ receptors in the rat semicircular canal results in excitatory modulation of calyx terminals (30).

Previous studies (5–7, 14) hypothesized that a change in the firing pattern interval histogram (IH_33_) may be linked to a GABAergic change. There are also studies that support GABA being intimately involved in anxiety behavior (15, 31). For example, in rats, intra-anterior cingulate cortex injections of GABA_A_R agonist have been shown to relieve anxiety-like behaviors (31). In a review study on GABA and anxiety (15), GABAergic interneurons in the amygdala were thought to play a key role in the modulation of anxiety responses both in normal and pathological states. Furthermore, medications such as S-valproate are known to improve anxious BD perhaps.

We used the firing pattern interval histogram, IH_33_, as a feature hypothesized to represent potential GABAergic and/or anxiety change. Our findings indicate that, in the BD populations, there are differences between those with and without anxiety disorder. We also looked at medication effects and found our model showing MS, AD, and AP medications potentially impacting the Diff_anx_ response.

Many anxiolytics act on GABA, for example, to increase the life of GABA in synapses (32). The locus coeruleus has bidirectional links to the vestibular nucleus (2). In the locus coeruleus, there are glial changes (33) linkable to glutamate signaling changes (33) also linkable (at least cortically) to GABA changes (34). While we have focused on GABA-related pathways, it is important not to exclude the impacts of other pathways which also will impact the responses of medicated subjects. Overall, as shown in Figures 3E–H, depressive severity can impact the encoding of anxiety disorder and vice-versa.

It is known that nearly all, if not all, the AP, AD, MS drugs listed in Supplementary Table S1 have some impact on anxiety symptomology. We recognize a major limitation herein that is the grouping of patients according to the medication regimen they are in. The medications within each group can have very different mechanisms of action, e.g., lithium and anti-epileptics may be classified as mood stabilizers and have different cellular targets and mechanisms of action. However, the bigger questions asked herein are as follows: 1. Did they as a medication group affect our anxiety feature(s) and; 2. Was this despite their individual modes of action? The answer to both questions appears to be yes at least for the MS medication group.

Previous EVestG studies hypothesized that the firing pattern (IH_33_) data were impacted by either alpha band activity or efferent vestibular system (EVS) modulation (5). While this remains plausible, the main frequency bands of Diff_anx_ difference in Figures 2, 3 and Supplementary Figure S1, i.e., approximately 6–13 Hz, lie within the same frequency range as hippocampal theta which has been linked to “anxiety circuits” as a biomarker (13). Hippocampal theta has been detected in the dorsal raphe nucleus of the vestibular system (35). Hippocampal theta may also be found in the vestibular periphery given that the vestibular nuclei (VN) have bidirectional projections from the dorsal raphe nuclei (36, 37), and the VN then projects to the vestibular periphery via the positive feedback looping efferent vestibular system (EVS) (38). Tai et al. have already noted that vestibular activity may be mediated by hippocampal theta (39). Critically, if hippocampal theta entrains the firing of any *peripheral* vestibular neurons, then EVestG may be detecting this entrainment. Such entrainment could be a biomarker of anxiety circuit changes.

The main limitation of this study is the sample size and, subsequently, the inability to fully isolate medication effects. A second limitation, as mentioned above, is the broad grouping of medications into three groups despite them having many individual and complex modes of action. A third limitation is the applied broad definition of anxiety disorder and lack of an instrument to determine the severity of that anxiety at baseline. A placebo-controlled study on a healthy group taking anxiolytics while being given a targeted anxiogenic stimulus is required and currently being undertaken. While beyond the scope of this study, a future study of the detailed impacts of neurotransmitter level changes on the EVS would help explain some of the results herein.

## 5 Conclusion

The main outcome is pilot data for a potentially clinically relevant baseline anxiety disorder test/measure potentially linked to GABA pathways. A secondary outcome is a potentially improved understanding of the physiology behind the depressive severity-based differences between the BD anxious and non-anxious measures. A future outcome could be a better reduction of the average pathological patient population comorbid anxiety level.

## Data availability statement

The original contributions presented in the study are included in the article/Supplementary material, further inquiries can be directed to the corresponding author.

## Ethics statement

The studies involving humans were approved by the Alfred Human Ethics Committee (Approval Number 95/06). The studies were conducted in accordance with the local legislation and institutional requirements. The participants provided their written informed consent to participate in this study. Written informed consent was obtained from the individual(s) for the publication of any potentially identifiable images or data included in this article.

## Author contributions

BL: Writing—original draft, Writing—review & editing. ZM: Writing—original draft, Writing—review & editing.

## Glossary

AD: on anti-depressant(s); notAD, not on anti-depressants can be on other medications
AP: anti-psychotic(s); notAP, not on anti-psychotics can be on other medications
All: on all combinations of medication groups (AD, AP, MS)
AD*AP: on anti-depressants and anti-psychotics, and not on mood stabilizers
AP*MS: on anti-psychotics and mood stabilizers, and not on anti-depressants
AD*MS: on anti-depressants and mood stabilizers, and not on anti-psychotics
AD*AP*MS: on anti-depressants and anti-psychotics and mood stabilizers
BD: bipolar disorder
Diff_anx_: the difference between anxious and non-anxious group responses
EVestG: evelectrovestibulography EVS, efferent vestibular system
F1, F2, F3, F4: Features 1, 2, 3, 4 (see Table 2 for definition)
FP: field potential
ICD: International Classification of Diseases
IH_33_: the time interval between each 33 detected field potentials
L1OCV: leave-one-out-cross-validation
LDA: linear discriminant analysis
MADRS: Montgomery–Asberg Depression Rating Scale
(M)ANCOVA: (Multiple) analysis of covariance
MDD: major depressive disorder
MINI: mini-international neuropsychiatric interview assessment
MMSE: mini-mental state exam
MS: mood stabilizer
Matched: matched for age, gender, and MADRS
NEER: neural event extraction routine
NM: Not on AP, AD, or MS medications
R: reduced symptomatic, MADRS <19
R&S: reduced and symptomatic populations combined
S: symptomatic, MADRS > 19
VN: vestibular nucleus
YMRS: Young Mania Rating Scale
