# Supplementary material for: Measuring anxiety disorder in bipolar disorder using EVestG: broad impact of medication groups
Source: Front Neurol. 2024 Jan 16;14:1303287. doi: 10.3389/fneur.2023.1303287 (PMC10824993; doi:10.3389/fneur.2023.1303287)
Supplement: Supplementary file 1 [file Data_Sheet_1.pdf]

Supplemental File:

Table S1. Study participant's demographics and psychiatric assessments. *BD subjects. The presence of Anxiety (A) and ADHD was based on the M.I.N.I. assessment and the Adult ADHD self-report scale respectively. ADHD subjects were taking Dextroamphetamine. Handedness: left (L), right (R), and both handed (B). S-valproate: sodium valproate.*

	**Table S1. BD study participants demographics and psychiatric assessments**												
**ID**	**diagnosis**	**Anxiety**	**Sex**	**Age**	**Hand**	**yrs since diagnosis**	**current antipsychotic**	**current antidepressant**	**current mood stabilizer**	**family history (M, P)**	**MMSE total**	**MADRS**	**YMRS**
178	BD-A type II		F	62	R	10		Mirtazapine	S-valproate	Y, N	29	6	10
236	BD-A type II	A	F	49	R	29	Quetiapine	Duloxetine	S-valproate	Y, Y	29	6	11
287	BD-A type II		F	44	R	20	Quetiapine		Lithium			5	2
172	BD-A type II		F	26	R	0	Quetiapine	Escitalopram oxalate		N, Y	29	4	0
325	BD-A type II		F	35	R	4	Quetiapine		Lithium		30	4	1
239	BD-A type II		M	65	R	8	Quetiapine	Venlafaxine	Lithium	Y, Y	28	4	4
111	BD-A type II		M	40	B	3			S-valproate	N, Y	29	4	11
231	BD-A type II	A	F	49	R	0		Desvenlafaxine		Y, N	30	3	0
248	BD-A type II		M	48	R	32			Lithium	N, Y	29	3	2
123	BD-A type II		M	62	R	40			S-valproate	N, Y	29	2	6
319	BD-A type II		M	43	R	7						2	10
297	BD-A type II		M	27	R	7	Amisulpride			Y, Y	30	1	0
253	BD-A type II		M	55	L	39			S-valproate, Lithium	Y, N	30	0	0
115	BD-A type II		F	62	L	32			Carbamazepine	Y, Y	30	0	4
89	BD-A type II		M	24	R	11	Olanzapine		S-valproate	N, Y	28	0	0
72	BD-A type II		M	75	R	7	Olanzapine	Citalopram	S-valproate	N, Y	25	0	0
**AVG**	**n=16**	**N=2**	**9M**	**47.88**		**15.56**					**28.93**	**2.75**	**3.81**
**STDEV**	**MADRS<=6**			**14.65**		**13.67**					**1.28**	**2.05**	**4.23**
324	BD-M (Type ?)	A	M	59	L	27	Quetiapine		Lithium	Y, N		18	8
63	BD-M type I		F	30	R	14	Aripiprazole			Y, N	30	17	0
201	BD-M type I	A	F	42	R	7	Quetiapine		Lithium		30	17	8
241	BD-M type II	A	F	45	R	22		Imipramine	S-valproate		27	17	13
315	BD-M type II		F	45	R	8		Escitalopram oxalate	S-valproate		30	14	10
286	BD-M type II	A	M	28	R	4	Ziprasidone	Duloxetine	S-valproate	Y, Y	30	13	1
247	BD-M type II	A	F	37	R	12	Aripiprazole	Duloxetine	Lithium, Lamotrigine		29	13	3
251	BD-M type II	A	F	48	R	12		Moclobemide	Lithuim	N, Y	29	11	11
95	BD-M type II		M	67	L	35	Quetiapine	Venlafaxine	S-valproate	Y, N	24	10	0
339	BD-M type II	A	F	33	L	7	Quetiapine	Venlafaxine	Carbamazepine		28	10	2
274	BD-M type II	A	F	27	R	6			Lithium	Y, N	29	10	11
158	BD-M type II		M	26	R	4		Venlafaxine		N, Y	29	10	0
245	BD-M type II	A	M	45	R	5				N, Y	30	9	3
210	BD-M type II		F	51	R	4		Paroxetine		N, Y	30	8	4
81	BD-M type I	A	M	63	R	35	Olanzapine	Sertraline	S-valproate, Lithium	N, Y	28	8	7
57	BD-M type I	A	M	35	R	21	Amisulpride		S-valproate, Lithium	N, Y	29	7	3
**AVG**	**n=16**	**N=11**	**7M**	**42.56**		**13.94**					**28.80**	**12.00**	**3.00**
**STDEV**	**all MADRS 7 to 19**			**12.42**		**10.46**					**1.56**	**3.54**	**4.07**
**AVG**	**n=32 (BD-R)**	**N=13**	**16M**	**45.22**		**14.75**					**28.86**	**7.38**	**4.53**
**STDEV**	**all MADRS <=19**			**13.84**		**12.20**					**1.43**	**5.45**	**4.32**
53	BD-S type I	A	F	49	R	9		Escitalopram oxalate		Y, N	30	42	0
258	BD-S type II		F	52	R	30		Venlafaxine		N, Y	30	37	3
56	BD-S type I	A	F	32	R	17		Sertraline, Reboxitine	Lamotrigine	Y, N	29	35	0
30	BD-S type I	A	F	54	R	16	Amisulpride	Duloxetine			28	34	0
167	BD-S type II	A	F	35	R	11	Aripiprazole	Citalopram		Y, Y	30	34	0
99	BD-S type I		F	60	R	10	Trifluoperazine	Sertraline	Lithium	Y, N	26	31	0
314	BD-S type II		M	59	R	26	Quetiapine	Mirtazapine		Y, N	26	29	2
318	BD-S type II	A	M	29	R	1	Olanzapine	Venlafaxine	Lithium	Y, N	29	29	8
151	BD-S type II		F	67	R	10	Quetiapine, Aripiprazole			N, Y	28	29	0
92	BD-S type I	A	M	41	R	2.5	Olanzapine, Quetiapine				28	27	0
122	BD-S type II		F	37	R	5		Fluoxetine	Carbamazepine		27	26	4
284	BD-S type II	A	F	39	R	20	Quetiapine		S-valproate	N, Y	30	26	11
85	BD-S type II	A	M	71	R	19		Venlafaxine	Lithium	Y, N	30	24	0
58	BD-S type I	A	M	59	L	34	Neulactol	Mirtazapine		Y, N	29	21	4
285	BD-S type II		F	55	R	32	Resperidone		S-valproate	Y, Y	28	21	6
261	BD-S type II		F	63	R	26		Sertraline		Y, Y	30	21	13
46	BD-S type I	A	F	45	R	27	Quetiapine	Doxepin	S-valproate, Lithium	N, Y	30	21	0
53b	BD-S type I	A	F	49	R	9		Escitalopram oxalate		Y, N	30	42	0
**AVG**	**n=18**	**N=11**	**5M**	**49.82**		**17.38**					**28.71**	**28.65**	**3.00**
**STDEV**	**MADRS >= 20**			**12.35**		**10.19**					**1.36**	**6.06**	**4.07**
**AVG**	**n=50**	**N=24**	**21M**	**46.82**		**15.66**					**28.80**	**14.76**	**4.00**
**STDEV**	**all MADRS**			**13.66**		**11.73**					**1.42**	**11.73**	**4.34**

Methods:

BGi segment selection

There were 5 background (BGi, no motion) segments analyzed for each participant (before the: upward translation, back tilt, ipsilateral tilt, contralateral tilt and rotation stimulus—the dynamic phases were not used in this study).

Normalization

As most classification techniques assume a normal distribution for the features used, all extracted features were tested for distribution normality using:

$Z = skew / SE_{skew}$ *and* $Z = kurtosis / SE_{kurtosis}$; $\alpha=0.05$, *Z test* > 1.96, *SE = standard error*

Medication compensation

For the BD population as only 2 BD subjects were not on medications the effect of medication was explored as follows. We tested for medication effects by combining subjects based on whether they were (or were not) on each drug group. This means, for example, the not-on-mood-stabilizer (**notMS**) medicated group could contain subjects on anti-psychotics (AP) or anti-depressants (AD) or AP&AD or no-medication (NM). The subjects were first grouped as **notMS**, **notAP**, **notAD** (Eq. 2-4) depending on the medication they were not taking, and these responses were evaluated and plotted (respectively orange, green, blue bars Figures 2 and S1). The total medicated anxious minus non-anxious ($Diff_{anx}$) response (‘**All**’ equals the $Diff_{anx}$ response with any combination of AP, AD, MS, NM) was the response of all 50 (anx=24, non-anx=26) BD subjects (i.e., black bars in Figures 2 and S1; Eq. 1 below). **Note**: this is the $Diff_{anx}$ response with the effect of medications superimposed exaggerating or suppressing the ($Diff_{anx}$) response. The difference between the with and without anxiety ($Diff_{anx}$) medicated response (black bars) and average response of each **NOT**-medicated group gives an indication of the effect each medication group might have. (Note: & is logical ‘AND’). The (sub)-populations are:

All = AP or MS or AD or AP&AD&AD&MS or AP&MS or AP&AD&MS or NM, Eq. (1)

notMS = AP or AD or AP&AD or NM, Eq. (2)

notAP = AD or MS or AD&MS or NM, Eq. (3)

notAD = AP or MS or AP&MS or NM, Eq. (4)

1. The impact of MS's on the $Diff_{anx}$ response was determined as: **All minus notMS** i.e., the difference between the orange (population without MS medications) and black (All) bars in Figures 2 and S1.
2. The impact of AP's on the $Diff_{anx}$ response was determined as: **All minus notAP** i.e., the difference between the green and black traces in Figures 2 and S1.
3. The impact of AD's on the $Diff_{anx}$ response was determined as: **All minus notAD** i.e., the difference between the blue and black traces in Figures 2 and S1.
4. The first estimated of the $Diff_{anx}$ response with medication impact removed (NoMed) is:
   All minus **notAP + All** minus **notAD + All** minus **notMS, (each component is weighted by the respective sample size).** However, this estimate includes the repeated effects of multiple medications i.e., 3**MS&AD&AP**, 2**MS&AP**, 2**MS&AD**, and 2**AD&AP;**
5. Lastly, a sample size weighted repeated multiple medication term is then subtracted from the first NoMed estimate) to produce a final offset which is added to the black bars in Fig. 2B to produce an indicator of the medication impact removed (red bars) $Diff_{anx}$ response in Figures 2 and S1.

These process steps (1-5) were then repeated for the R and S sub-populations as well as for their matched subgroups.

If the medication removed (red bars adjusted $p<0.0055$) and medicated (black bars, $p<0.05$) confidence interval ranges overlapped, the medication effect was considered non-significant. Note the R and S plots of Fig. S1 are shape-wise almost the reverse of each other on both the left and right sides.

To make this $Diff_{anx}$ medication removed adjustment, for each anxious subject, each feature bin value analyzed was offset by the difference between the medicated and non-medicated bin values. Statistical analysis of these

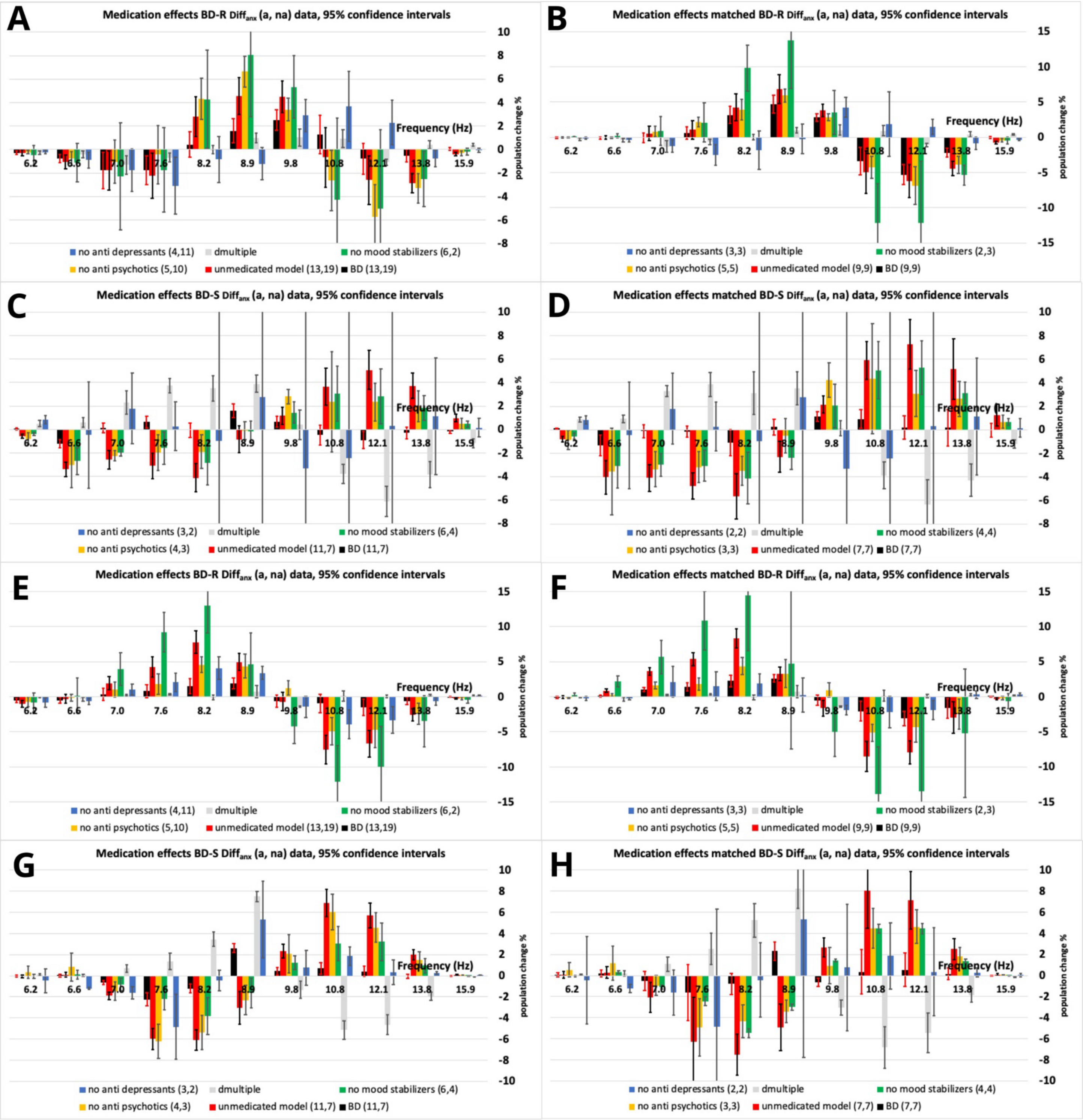

Figure S1. Medication effects on BD R and S sub populations. A-D left side, E-H right side. (S&R population data is shown in paper Fig. 3A-D)

‘medication removed’ responses use an adjusted p=0.0055. We now statistically compare the medication removed and medicated population responses.

Table S2 shows the BD population statistical analysis results. For each population (S&R, R, S) the analysis is performed for the entire populations, matched (age, gender, MADRS) subpopulations and a medication removed estimate of each. Red text indicates significance. Highlighted text indicates caution as another variable e.g., Age has a significant effect or there is a potential test violation e.g., Lavene’s Test. See main paper for discussion of statistically significant findings.

Table S2a. BD S&R $Diff_{anx}$ data

	‘All’ left	‘matched’ left	Medication ‘removed‘ ‘All’ left	Medication ‘removed’ ‘matched’ left
**N = NA, A**	26, 24	18, 18	26, 24	18, 18
**ROC**	0.654	0.734	0.721	0.797
**ANCOVA**	**Univariate:** F3 not significant	**Univariate:** F3 not significant	**Univariate:** F4	**Univariate:** F4
**CV=age, MADRS; FF=feature, gender**	F(1,44)=2.317	F(1,26)=3.374	F(1,44)=25.462	F(1,26)=14.313
Sig, η^2, power	0.135, 0.050, 0.319	0.078, 0.115, 0.425	<0.001, 0.367, 0.999	<0.001, 0.355, 0.954
**Significant Between-subjects effects**			**MADRS:** F(1,42)=4.057	
Sig, η^2, power			0.050, 0.088, 0.503	

Table S2b. BD R $Diff_{anx}$ data

	‘All’	‘matched’	Medication ‘removed’ ‘All’	Medication ‘removed’ ‘matched’
**N = NA, A**	19, 13	9, 9	19, 13	9, 9
**ROC**	F3=0.704 F1=0.664	F3=0.864 F1=0.642	F3=0.777 F4=0.972	F3=0.926 F4=1.0
**MANCOVA**	**Multivariate:** F3, F1 not signif.	**Multivariate:** F3, F1.	**Multivariate:** F3, F4	**Multiivariate:** F3, F4
**CV=age, MADRS; FF=feature(s), gender**	F(2,25)=1.282 Wilkes-λ=0.252	F(2,11)=5.133 Wilkes-λ=0.517	F(2,25)=30.497 Wilkes-λ=0.291	F(2,11)=17.168 Wilkes-λ=0.243
Sig, η^2, power	0.295, 0.093, 0.319	0.027, 0.483, 0.702	<0.001, 0.709, 1.0	<0.001, 0.757, 0.997
**Significant Between-subjects effects**	**Age:** F(1,24)=5.335			
Sig, η^2, power	0.030, 0.532, 0.601			
	**Univariate:** F3 not significant	**Univariate:** F3	**Univariate:** F3	**Univariate:** F3
**CV=age, MADRS; FF=feature, gender**	F(1,26)=2.543	F(1,12)=10.643	F(1,26)=4.873	F(1,12)=18.788
Sig, η^2, power	0.123, 0.0890.336	0.007, 0.470, 0.849	0.036, 0.158, 0.566	<0.001, 0.610, 0.978
	**Univariate:** F1 not significant	**Univariate:** F1 not significant	**Univariate:** F4	**Univariate:** F4
**CV=age, MADRS; FF=feature, gender**	F(1,26)=0.433	F(1,12)=0.093	F(1,26)=48.826	F(1,12)=29.669
Sig, η^2, power	0.516, 0.016, 0.097	0.765, 0.008, 0.059	<0.001, 0.653, 1.0	<0.001, 0.712, 0.999

Table S2c. BD S $Diff_{anx}$ data

	‘All’	‘matched’	Medication ‘removed’ ‘All’	Medication ‘removed’ ‘matched’
**N = NA, A**	7, 11	7, 7	7, 11	7, 7
**ROC**	F1=0.669 F2=0.610	F1=0.592 F2=0.653	F3=0.623 F4=0.909	F3=0.653 F4=0.878
**Linear Regression (LR)**	F1, F2: R=0.607 F(5,12)=1.40	F1, F2: R=0.538 F(5,8)=0.653	F3, F4: R=0.759 F(5,12)=3.257	F3, F4: R=0.724 F(5,8)=1.758
**(non-normal distributions)**	sig=0.292	sig=0.669	sig=0.044	sig=0.228
**Non-Parametric Quade ANCOVA Test**	**Quade:** F1 not significant	**Quade:** F1 not significant	**Quade:** F3 not significant	**Quade:** F3 not significant
**(non-normal distributions)**				
**CV=age, MADRS, gender**	F(1,16)=0.597	F(1,12)=0.054	F(1,16)=0.733	F(1,12)=0.626
Sig, t	0.451, -0.772	0.820, 0.232	0.405, 0.856	0.444, 0.792
	**Quade:** F2 not significant	**Quade:** F2 not significant	**Quade:** F4	**Quade:** F4
**CV=age, MADRS, gender**	F(1,16)=0.162	F(1,12)=0.753	F(1,16)=9.244	F(1,12)=8.904
Sig, t	0.692, 0.403	0.403, -0.868	0.008, 3.040	0.011, 2.984
